# Bis(2-hydroxy­benzaldehyde oximato-κ*O*)triphenyl­anti­mony(V)

**DOI:** 10.1107/S1600536809043542

**Published:** 2009-10-28

**Authors:** Lei Dong, Handong Yin, Liyuan Wen, Daqi Wang

**Affiliations:** a College of Chemistry and Chemical Engineering, Liaocheng University, Shandong 252059, People’s Republic of China

## Abstract

The mol­ecule of the title compound, [Sb(C_6_H_5_)_3_(C_7_H_6_NO_2_)_2_], is located on a twofold axis defined by the metal center and two C atoms of a coordinated phenyl group. The Sb center has a slightly distorted trigonal-bipyramidal geometry, with the axial positions occupied by the O atoms of symmetry-related 2-hydroxy­benzaldehyde oximate ligands. An intra­molecular O—H⋯N inter­action is present. The crystal structure is stabilized by C—H⋯O inter­actions.

## Related literature

For related structures, see: Wang *et al.* (2004 ▶); Sharutin *et al.* (2004 ▶).
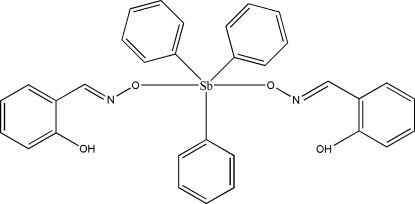


## Experimental

### 

#### Crystal data

[Sb(C_6_H_5_)_3_(C_7_H_6_NO_2_)_2_]
*M* *_r_* = 625.31
Tetragonal, 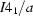

*a* = 16.6012 (15) Å
*c* = 20.775 (2) Å
*V* = 5725.6 (9) Å^3^
*Z* = 8
Mo *K*α radiation
μ = 1.00 mm^−1^
*T* = 298 K
0.38 × 0.30 × 0.17 mm

#### Data collection

Siemens SMART CCD area-detector diffractometer
Absorption correction: multi-scan (*SADABS*; Sheldrick, 1996 ▶) *T*_min_ = 0.702, *T*_max_ = 0.848
17411 measured reflections
3487 independent reflections
2410 reflections with *I* > 2σ(*I*)
*R*_int_ = 0.034

#### Refinement

*R*[*F* ^2^ > 2σ(*F* ^2^)] = 0.031
*wR*(*F* ^2^) = 0.085
*S* = 1.09
3487 reflections
178 parameters
H-atom parameters constrained
Δρ_max_ = 1.38 e Å^−3^
Δρ_min_ = −0.68 e Å^−3^

### 

Data collection: *SMART* (Siemens, 1996 ▶); cell refinement: *SAINT* (Siemens, 1996 ▶); data reduction: *SAINT*; program(s) used to solve structure: *SHELXS97* (Sheldrick, 2008 ▶); program(s) used to refine structure: *SHELXL97* (Sheldrick, 2008 ▶); molecular graphics: *SHELXTL* (Sheldrick, 2008 ▶); software used to prepare material for publication: *SHELXTL*.

## Supplementary Material

Crystal structure: contains datablocks I, global. DOI: 10.1107/S1600536809043542/gk2230sup1.cif

Structure factors: contains datablocks I. DOI: 10.1107/S1600536809043542/gk2230Isup2.hkl

Additional supplementary materials:  crystallographic information; 3D view; checkCIF report

## Figures and Tables

**Table 1:** Hydrogen-bond geometry (Å, °)

*D*—H⋯*A*	*D*—H	H⋯*A*	*D*⋯*A*	*D*—H⋯*A*
C7—H7⋯O2^i^	0.93	2.58	3.294 (5)	134
O2—H2⋯N1	0.82	1.89	2.617 (4)	146

Symmetry code: (i) 
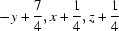
.

**Fig. 1.**
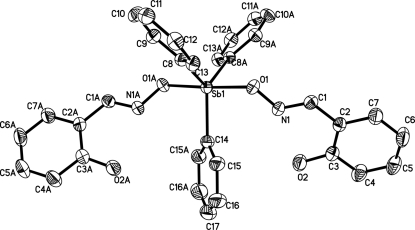
The molecular structure of the compound, showing 50% probability displacement ellipsoids. H atoms have been omitted for clarity. Symmetry code: (A) = -x + 1, -y + 3/2, z.

**Fig. 2.**
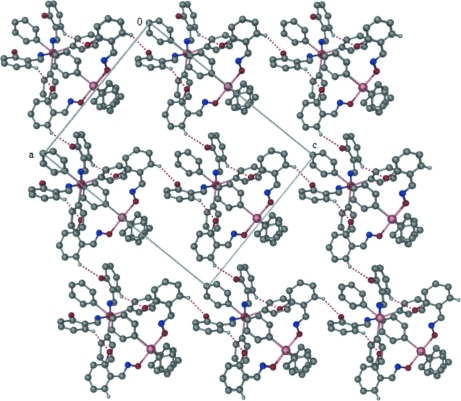
View of the two-dimensional layer structure in the title compound. Intermolecular hydrogen bonds are shown as dashed lines.

## supplementary crystallographic information

### Comment

Among numerous multidentate organic ligands, oximes are of particular importance, because of their remarkable structural diversity and biological implications. As a continuation of our interest in this area, we have synthesized the title complex. The molecular structures of the compound is depicted in Fig.1. The antimony atom in the compound is five-coordinate, and its geometries is represented as a distorted trigonal bipyramid. The Sb—O bond distance of 2.078 (2) Å is similar to that reported by Wang  (Wang *et al.*, 2004; 2.094 (2)Å) . The Sb—C bond distances [2.102 (3) Å; 2.097 (5) Å] fall in the normal range for Sb—*C*(phenyl) bonds. In the crystal structure, molecules are linked by C—H···O hydrogen bonds.

### Experimental

Salicylaldoxime (0.4 mmol) was added to a methanol solution of sodium (0.4 mmol) and heated under reflux for 0.5 h. To this solution was added triphenylantimony dichloride (0.2 mmol) in benzene and the mixture was refluxed for 5 h, cooled and filtered. The filtrate was evaporated *in vacuo*. The obtained solid was recrystallized from dichloromethane-petroleum ether. (yield 86%. m.p.431k). Anal. Calcd (%) for C_32_H_27_N_2_O_4_Sb (Mr = 625.33): C, 61.46; H, 4.35; N, 4.48; O,10.23. Found (%): C, 61.47; H, 4.40; N, 4.46; O,10.26.

### Refinement

The C—H and O—H H atoms were positioned with idealized geometry (O—H = 0.82 Å and C—H = 0.93 Å) and were refined using a riding model approximation with  U_iso_(H) = 1.2 U_eq_(C, O).The final difference map showed a high residual peak (1.38 e Å^-3^) at a distance of 0.35 Å from H17.

### Crystal data

**Table:**

[Sb(C_6_H_5_)_3_(C_7_H_6_NO_2_)_2_]	*D*_x_ = 1.451 Mg m^−^^3^
*M**_r_* = 625.31	Mo *K*α radiation, λ = 0.71073 Å
Tetragonal, *I*4_1_/*a*	Cell parameters from 5826 reflections
Hall symbol: -I 4ad	θ = 2.5–25.5°
*a* = 16.6012 (15) Å	µ = 1.00 mm^−^^1^
*c* = 20.775 (2) Å	*T* = 298 K
*V* = 5725.6 (9) Å^3^	Block, colourless
*Z* = 8	0.38 × 0.30 × 0.17 mm
*F*(000) = 2528	

### Data collection

**Table:**

Siemens SMART CCD area-detector diffractometer	3487 independent reflections
Radiation source: fine-focus sealed tube	2410 reflections with *I* > 2σ(*I*)
graphite	*R*_int_ = 0.034
φ and ω scans	θ_max_ = 28.3°, θ_min_ = 1.6°
Absorption correction: multi-scan (*SADABS*; Sheldrick, 1996)	*h* = −21→21
*T*_min_ = 0.702, *T*_max_ = 0.848	*k* = −9→22
17411 measured reflections	*l* = −26→26

### Refinement

**Table:**

Refinement on *F*^2^	Primary atom site location: structure-invariant direct methods
Least-squares matrix: full	Secondary atom site location: difference Fourier map
*R*[*F*^2^ > 2σ(*F*^2^)] = 0.031	Hydrogen site location: inferred from neighbouring sites
*wR*(*F*^2^) = 0.085	H-atom parameters constrained
*S* = 1.09	*w* = 1/[σ^2^(*F*_o_^2^) + (0.030*P*)^2^ + 9.4858*P*] where *P* = (*F*_o_^2^ + 2*F*_c_^2^)/3
3487 reflections	(Δ/σ)_max_ = 0.001
178 parameters	Δρ_max_ = 1.38 e Å^−^^3^
0 restraints	Δρ_min_ = −0.68 e Å^−^^3^

### Fractional atomic coordinates and isotropic or equivalent isotropic displacement parameters (Å^2^)

**Table:**

	*x*	*y*	*z*	*U*_iso_*/*U*_eq_	
Sb1	0.5000	0.7500	0.977300 (13)	0.03630 (10)	
N1	0.65745 (16)	0.80646 (15)	0.93840 (13)	0.0438 (6)	
O1	0.62512 (13)	0.74876 (13)	0.97978 (10)	0.0442 (5)	
O2	0.66631 (16)	0.89097 (18)	0.83231 (13)	0.0716 (8)	
H2	0.6454	0.8611	0.8589	0.107*	
C1	0.7300 (2)	0.8254 (2)	0.95055 (16)	0.0474 (8)	
H1	0.7551	0.8043	0.9869	0.057*	
C2	0.7748 (2)	0.8797 (2)	0.90850 (17)	0.0467 (8)	
C3	0.7419 (2)	0.9098 (2)	0.85145 (18)	0.0515 (8)	
C4	0.7874 (3)	0.9596 (2)	0.8126 (2)	0.0704 (11)	
H4	0.7653	0.9799	0.7747	0.084*	
C5	0.8650 (3)	0.9796 (3)	0.8293 (2)	0.0762 (13)	
H5	0.8951	1.0130	0.8026	0.091*	
C6	0.8981 (3)	0.9506 (3)	0.8848 (2)	0.0778 (13)	
H6	0.9506	0.9643	0.8959	0.093*	
C7	0.8535 (2)	0.9012 (3)	0.92426 (19)	0.0647 (10)	
H7	0.8763	0.8819	0.9621	0.078*	
C8	0.50453 (19)	0.64233 (17)	1.03043 (14)	0.0384 (7)	
C9	0.4426 (2)	0.62433 (19)	1.07230 (15)	0.0477 (8)	
H9	0.3979	0.6579	1.0751	0.057*	
C10	0.4475 (3)	0.5556 (2)	1.11027 (17)	0.0573 (9)	
H10	0.4062	0.5435	1.1389	0.069*	
C11	0.5130 (3)	0.5054 (2)	1.10563 (17)	0.0572 (9)	
H11	0.5159	0.4596	1.1313	0.069*	
C12	0.5738 (2)	0.5225 (2)	1.06345 (18)	0.0543 (9)	
H12	0.6178	0.4880	1.0603	0.065*	
C13	0.5701 (2)	0.59106 (19)	1.02534 (16)	0.0458 (8)	
H13	0.6114	0.6025	0.9965	0.055*	
C14	0.5000	0.7500	0.8764 (2)	0.0523 (12)	
C15	0.5554 (3)	0.7042 (2)	0.84211 (19)	0.0655 (11)	
H15	0.5931	0.6734	0.8643	0.079*	
C16	0.5554 (4)	0.7039 (4)	0.7751 (2)	0.0985 (19)	
H16	0.5922	0.6729	0.7522	0.118*	
C17	0.5000	0.7500	0.7441 (4)	0.102 (3)	
H17	0.5000	0.7500	0.6993	0.123*	

### Atomic displacement parameters (Å^2^)

**Table:**

	*U*^11^	*U*^22^	*U*^33^	*U*^12^	*U*^13^	*U*^23^
Sb1	0.03787 (17)	0.03277 (16)	0.03825 (16)	0.00025 (13)	0.000	0.000
N1	0.0412 (15)	0.0414 (14)	0.0487 (15)	−0.0016 (12)	0.0064 (12)	0.0021 (12)
O1	0.0393 (12)	0.0429 (12)	0.0506 (13)	0.0003 (10)	0.0072 (10)	0.0083 (10)
O2	0.0554 (16)	0.093 (2)	0.0665 (17)	−0.0152 (15)	−0.0083 (13)	0.0257 (15)
C1	0.0455 (19)	0.0501 (19)	0.0466 (18)	0.0021 (15)	0.0034 (15)	0.0023 (16)
C2	0.0446 (18)	0.0428 (18)	0.053 (2)	−0.0002 (15)	0.0048 (16)	−0.0019 (16)
C3	0.049 (2)	0.048 (2)	0.057 (2)	−0.0017 (16)	0.0054 (17)	0.0032 (17)
C4	0.067 (3)	0.071 (3)	0.074 (3)	−0.005 (2)	0.005 (2)	0.026 (2)
C5	0.068 (3)	0.068 (3)	0.093 (3)	−0.016 (2)	0.015 (2)	0.022 (2)
C6	0.055 (2)	0.082 (3)	0.096 (3)	−0.023 (2)	0.004 (2)	0.014 (3)
C7	0.054 (2)	0.075 (3)	0.065 (2)	−0.009 (2)	−0.0021 (19)	0.011 (2)
C8	0.0441 (17)	0.0323 (15)	0.0387 (16)	−0.0002 (13)	−0.0010 (13)	−0.0017 (13)
C9	0.052 (2)	0.0418 (18)	0.0489 (19)	0.0026 (15)	0.0063 (16)	−0.0012 (15)
C10	0.074 (3)	0.0462 (19)	0.052 (2)	−0.0065 (18)	0.0121 (18)	0.0057 (16)
C11	0.080 (3)	0.0347 (17)	0.057 (2)	−0.0026 (18)	−0.007 (2)	0.0049 (16)
C12	0.059 (2)	0.0369 (17)	0.067 (2)	0.0070 (16)	−0.0069 (19)	−0.0009 (17)
C13	0.0474 (19)	0.0372 (17)	0.0527 (19)	0.0004 (14)	0.0028 (16)	−0.0015 (15)
C14	0.069 (3)	0.048 (3)	0.041 (2)	−0.015 (2)	0.000	0.000
C15	0.079 (3)	0.064 (2)	0.054 (2)	−0.015 (2)	0.010 (2)	−0.0095 (19)
C16	0.127 (5)	0.105 (4)	0.064 (3)	−0.044 (4)	0.027 (3)	−0.030 (3)
C17	0.141 (8)	0.110 (7)	0.055 (4)	−0.065 (6)	0.000	0.000

### Geometric parameters (Å, °)

**Table:**

Sb1—O1^i^	2.078 (2)	C7—H7	0.9300
Sb1—O1	2.078 (2)	C8—C9	1.379 (4)
Sb1—C14	2.097 (5)	C8—C13	1.386 (4)
Sb1—C8^i^	2.102 (3)	C9—C10	1.390 (5)
Sb1—C8	2.102 (3)	C9—H9	0.9300
N1—C1	1.271 (4)	C10—C11	1.373 (5)
N1—O1	1.394 (3)	C10—H10	0.9300
O2—C3	1.353 (4)	C11—C12	1.367 (5)
O2—H2	0.8200	C11—H11	0.9300
C1—C2	1.459 (5)	C12—C13	1.388 (5)
C1—H1	0.9300	C12—H12	0.9300
C2—C7	1.394 (5)	C13—H13	0.9300
C2—C3	1.397 (5)	C14—C15^i^	1.389 (5)
C3—C4	1.381 (5)	C14—C15	1.389 (5)
C4—C5	1.375 (6)	C15—C16	1.393 (6)
C4—H4	0.9300	C15—H15	0.9300
C5—C6	1.365 (6)	C16—C17	1.359 (7)
C5—H5	0.9300	C16—H16	0.9300
C6—C7	1.375 (5)	C17—C16^i^	1.359 (7)
C6—H6	0.9300	C17—H17	0.9300
			
O1^i^—Sb1—O1	177.16 (12)	C6—C7—H7	119.4
O1^i^—Sb1—C14	91.42 (6)	C2—C7—H7	119.4
O1—Sb1—C14	91.42 (6)	C9—C8—C13	120.0 (3)
O1^i^—Sb1—C8^i^	86.72 (10)	C9—C8—Sb1	119.3 (2)
O1—Sb1—C8^i^	91.79 (10)	C13—C8—Sb1	120.7 (2)
C14—Sb1—C8^i^	121.67 (8)	C8—C9—C10	119.5 (3)
O1^i^—Sb1—C8	91.79 (10)	C8—C9—H9	120.2
O1—Sb1—C8	86.72 (10)	C10—C9—H9	120.2
C14—Sb1—C8	121.67 (8)	C11—C10—C9	120.3 (3)
C8^i^—Sb1—C8	116.65 (16)	C11—C10—H10	119.9
C1—N1—O1	114.4 (3)	C9—C10—H10	119.9
N1—O1—Sb1	111.27 (16)	C12—C11—C10	120.3 (3)
C3—O2—H2	109.5	C12—C11—H11	119.9
N1—C1—C2	121.1 (3)	C10—C11—H11	119.9
N1—C1—H1	119.4	C11—C12—C13	120.2 (3)
C2—C1—H1	119.4	C11—C12—H12	119.9
C7—C2—C3	118.3 (3)	C13—C12—H12	119.9
C7—C2—C1	119.7 (3)	C8—C13—C12	119.7 (3)
C3—C2—C1	122.0 (3)	C8—C13—H13	120.2
O2—C3—C4	118.3 (3)	C12—C13—H13	120.2
O2—C3—C2	122.0 (3)	C15^i^—C14—C15	118.4 (5)
C4—C3—C2	119.8 (4)	C15^i^—C14—Sb1	120.8 (2)
C5—C4—C3	120.6 (4)	C15—C14—Sb1	120.8 (2)
C5—C4—H4	119.7	C14—C15—C16	121.0 (5)
C3—C4—H4	119.7	C14—C15—H15	119.5
C6—C5—C4	120.3 (4)	C16—C15—H15	119.5
C6—C5—H5	119.8	C17—C16—C15	118.1 (6)
C4—C5—H5	119.8	C17—C16—H16	120.9
C5—C6—C7	119.9 (4)	C15—C16—H16	120.9
C5—C6—H6	120.1	C16^i^—C17—C16	123.4 (7)
C7—C6—H6	120.1	C16^i^—C17—H17	118.3
C6—C7—C2	121.1 (4)	C16—C17—H17	118.3
			
C1—N1—O1—Sb1	160.6 (2)	O1—Sb1—C8—C13	−27.7 (2)
O1^i^—Sb1—O1—N1	−132.17 (17)	C14—Sb1—C8—C13	61.9 (3)
C14—Sb1—O1—N1	47.83 (17)	C8^i^—Sb1—C8—C13	−118.1 (3)
C8^i^—Sb1—O1—N1	−73.93 (19)	C13—C8—C9—C10	1.5 (5)
C8—Sb1—O1—N1	169.48 (19)	Sb1—C8—C9—C10	−176.3 (3)
O1—N1—C1—C2	174.7 (3)	C8—C9—C10—C11	−0.8 (5)
N1—C1—C2—C7	178.1 (3)	C9—C10—C11—C12	−0.3 (6)
N1—C1—C2—C3	−3.6 (5)	C10—C11—C12—C13	0.5 (6)
C7—C2—C3—O2	179.2 (3)	C9—C8—C13—C12	−1.3 (5)
C1—C2—C3—O2	0.8 (5)	Sb1—C8—C13—C12	176.5 (2)
C7—C2—C3—C4	−0.2 (5)	C11—C12—C13—C8	0.2 (5)
C1—C2—C3—C4	−178.6 (3)	O1^i^—Sb1—C14—C15^i^	38.99 (19)
O2—C3—C4—C5	−179.0 (4)	O1—Sb1—C14—C15^i^	−141.01 (19)
C2—C3—C4—C5	0.4 (6)	C8^i^—Sb1—C14—C15^i^	−48.0 (2)
C3—C4—C5—C6	−0.3 (7)	C8—Sb1—C14—C15^i^	132.0 (2)
C4—C5—C6—C7	0.0 (7)	O1^i^—Sb1—C14—C15	−141.01 (19)
C5—C6—C7—C2	0.2 (7)	O1—Sb1—C14—C15	38.99 (19)
C3—C2—C7—C6	−0.1 (6)	C8^i^—Sb1—C14—C15	132.0 (2)
C1—C2—C7—C6	178.3 (4)	C8—Sb1—C14—C15	−48.0 (2)
O1^i^—Sb1—C8—C9	−27.5 (3)	C15^i^—C14—C15—C16	−0.3 (3)
O1—Sb1—C8—C9	150.1 (3)	Sb1—C14—C15—C16	179.7 (3)
C14—Sb1—C8—C9	−120.2 (2)	C14—C15—C16—C17	0.5 (6)
C8^i^—Sb1—C8—C9	59.8 (2)	C15—C16—C17—C16^i^	−0.2 (3)
O1^i^—Sb1—C8—C13	154.7 (2)		

Symmetry codes: (i) −*x*+1, −*y*+3/2, *z*.

### Hydrogen-bond geometry (Å, °)

**Table:**

*D*—H···*A*	*D*—H	H···*A*	*D*···*A*	*D*—H···*A*
C7—H7···O2^ii^	0.93	2.58	3.294 (5)	134
O2—H2···N1	0.82	1.89	2.617 (4)	146

Symmetry codes: (ii) −*y*+7/4, *x*+1/4, *z*+1/4.
